# Regulation of the Function of T Follicular Helper Cells and B Cells in Type 1 Diabetes Mellitus by the OX40/OX40L Axis

**DOI:** 10.1210/clinem/dgae248

**Published:** 2024-04-16

**Authors:** Xuan Du, Yan Zhu, Wen Lu, Nannan Fu, Qin Wang, Bimin Shi

**Affiliations:** Department of Endocrinology and Metabolism, The First Affiliated Hospital of Soochow University, Suzhou 215129, Jiangsu, China; Department of Endocrinology and Metabolism, The First Affiliated Hospital of Soochow University, Suzhou 215129, Jiangsu, China; Department of Endocrinology and Metabolism, The First Affiliated Hospital of Soochow University, Suzhou 215129, Jiangsu, China; Department of Immunology, Suzhou Medical College of Soochow University, Suzhou 215123, Jiangsu, China; Department of Immunology, Suzhou Medical College of Soochow University, Suzhou 215123, Jiangsu, China; Department of Endocrinology and Metabolism, The First Affiliated Hospital of Soochow University, Suzhou 215129, Jiangsu, China

**Keywords:** T1DM, Tfh, OX40, OX40L

## Abstract

**Objective/Main Outcome:**

To study the expression of OX40 on T follicular helper (Tfh) cells and the ligand OX40L on antigen-presenting cells (APCs) in peripheral blood of patients with type 1 diabetes mellitus (T1DM) and the role of OX40 signaling in promoting Tfh cells to assist B-cell differentiation.

**Design:**

Cross-sectional study.

**Setting:**

Endocrinology department of a university hospital.

**Participants:**

Twenty-five patients with T1DM and 35 with newly diagnosed type 2 diabetes mellitus (T2DM) from January 2021 to December 2021 (39 males, 21 females; mean age: 31.0 ± 4.5, range: 19-46 years).

**Interventions:**

None.

**Methods:**

The peripheral blood proportion of CD4^+^CD25^−^CD127^+^CXCR5^+^PD1^+^ Tfh cells in patients with T1DM or T2DM and the OX40L expression in CD14^+^ monocytes and CD19^+^ B cells were analyzed by flow cytometry. The OX40 signal effect on Tfh-cell function was analyzed by coincubating B cells with Tfh cells under different conditions. Flow cytometry detected the ratio of CD19^−^CD138^+^ plasmacytes.

**Results:**

The Tfh cells ratio and intracellular IL-21 expression in peripheral blood was significantly higher in patients with T1DM than with T2DM, and the OX40 expression in peripheral Tfh cells and OX40L expression in APC were significantly higher in T1DM. After adding OX40L protein, the CD19^−^CD138^+^-plasmacytes percentage was significantly increased and higher in T1DM. Blocking of anti-OX40L monoclonal antibodies significantly reduced the plasmacytes ratio.

**Conclusion:**

The peripheral Tfh cells proportion increased and the OX40 expression in peripheral Tfh cells was upregulated in patients with T1DM vs patients with T2DM. OX40/OX40L signaling enhanced the Tfh-cell function to assist B-cell differentiation, which may contribute to the pathogenesis of T1DM.

Type 1 diabetes mellitus (T1DM) is an organ-specific chronic autoimmune disease. Characterized by insulin deficiency due to islet-β cell destruction, T1DM patients depend on exogenous insulin to maintain glucose metabolism (1, 2). Although environmental factors are implicated, immunological dysfunction has a crucial role in the pathogenesis of T1DM. However, the exact mechanisms of autoimmune responses remain unclear. Abnormal functions of T follicular helper (Tfh) and B cells have important roles in the development of T1DM. The differentiation process of B cells requires the help of Tfh cells, which are important regulators of humoral immune responses. The inflammatory cytokine IL-21 secreted by Tfh cells promotes activation of B cells and secretion of other cytokines, such as IL-17, thereby aggravating inflammation. In peripheral lymphoid tissues, following activation by dendritic cells (DCs), naïve T cells rapidly upregulate CXCR5 and arrive at the T-cell–B-cell border under the chemotaxis of CXCL13 secreted by B cells and then enter the lymphoid follicle to become Tfh cells, which help naïve B cells differentiate into plasma cells by secreting IL-21 and help B cells produce antigen-specific antibodies to induce humoral immune reponses (3-5). Hence, enhancement of Tfh cells may have an important role in the pathogenesis of T1DM.

OX40 molecules are mainly expressed on the membranes of activated CD4^+^ and CD8^+^ T cells. Synergizing with ICOS/ICOSL, OX40/OX40L signaling induces activation of antigen-specific CD4^+^ T cells and upregulates CXCR5 expression, which promotes T-cell migration to lymphoid follicles and germinal centers (GC) formation (6-8). As an independent subset of CD4^+^ T cells, Tfh cells have been confirmed to also express OX40 molecules. However, there are no published reports on OX40^+^ Tfh cells in T1DM. Furthermore, the potential mechanisms underlying the function of OX40^+^ Tfh cells in T1DM have not been elucidated.

Our study aimed to detect the expression of OX40/OX40L and Tfh cells in the peripheral blood of patients with T1DM and explore the potential mechanisms of Tfh and B cell differentiation regulated by OX40/OX40L signaling in T1DM, which could provide potential therapeutic targets for developing novel therapies for T1DM.

## Materials and Methods

### Patients and Laboratory Analysis

A total of 25 patients living with T1DM and 35 patients with newly diagnosed T2DM between January 2021 and December 2021, including 39 males and 21 females (mean age: 31.0 ± 4.5 years, range: 19-46 years), in the Department of Endocrinology of the First Affiliated Hospital of Soochow University were included in this cross-sectional study. The study was approved by the Ethics Committee of the First Affiliated Hospital of Soochow University (Suzhou, China). Patients and controls provided written informed consent. The diagnostic criteria for T1DM were according to the the T1DM Exchange Clinic Registry, and the patients with T2DM were diagnosed according to the 1999 World Health Organization diagnostic criteria (9, 10). Patients with secondary diabetes, acute or chronic inflammatory diseases, infectious diseases, additional autoimmune disorders, and cancers were excluded. In addition, body height and weight were measured. Based on the measurements, the body mass index was calculated. Blood samples from an antecubital vein were collected in a quiet state in the morning after fasting overnight for 12 hours, and fasting blood glucose, glycosylated hemoglobin A1c (HbA1c), C-peptide, C-peptide after a 2-h meal (2hCP), glutamic acid decarboxylase antibodies (GADA), zinc transporter-8 autoantibodies (ZnT8A), islet-cell antibodies (ICA), and insulin autoantibody (IAA) levels were measured. The glucose oxidation method was used to measure the blood glucose levels, with an automatic biochemical analyzer (7600, Hitachi Company, Japan). HbA1c was measured by high-performance liquid chromatography and a Bio-Rad Variant II analyzer. (HLC-723G8, TOSOH Company, Japan). Serum C and 2hCP levels were measured by chemiluminescent immunoassay (AIA-2000ST, TOSOH Company). GADA, ZnT8A, ICA, and IAA (iFLASH test panel, YHLO Company, China) levels were measured by chemiluminescence immunoassay analyzer (iFLASH 3000, YHLO Company).

### Cell Staining and Flow Cytometry

To assess the expression of OX40 on Tfh cells and OX40L on CD14^+^ monocytes and CD19^+^ B cells, heparinized peripheral venous blood was obtained from the study subjects. Flow cytometry (Beckman Coulter, USA) was performed on peripheral blood mononuclear cells (PBMCs), separated by centrifugation of blood over a Ficoll–Conray gradient (Dayou Co, China) and incubated with fluorochrome-labeled monoclonal antibodies (mAbs) for 30 minutes. Anti-CD4-BV605 (catalog no. 300556, RRID: AB_2564391), anti-CD25-BV421 (catalog no. 562442, RRID: AB_11154578), anti-PD1-FITC (catalog no. 557860, RRID: AB_2159176), anti-OX40-Percpcy5.5 (catalog no. 350010, RRID: AB_10719224), anti-CD19-BV605 (catalog no. 562653, RRID: AB_2722592), and anti-OX40L-PE (catalog no. 558164, RRID: AB_647195) mAbs were obtained from Becton Dickinson, USA. Anti-CXCR5-APC (catalog no. 12-9185-42, RRID: AB_11219877) was obtained from eBioscience, USA, and anti-CD14-PEvivo-770 was obtained from Miltenyi Biotec, Germany.

To detect intracellular IL-21 produced by Tfh cells, PBMCs isolated from diabetes mellitus patients were cultured with anti-CD3 mAb (200 ng/mL, catalog no. BE0001-2, RRID: AB_1107632, Biocell, USA) at 37°C, 5% CO_2_, and 95% humidity. The next day, phorbol-12-myristate-13-acetate (50 µg/mL, Sigma-Aldrich, USA), ionomycin (750 ng/mL, BioVision, USA), and GolgiStopTM (catalog no. 51-2092KZ, RRID: AB_2869009, BD, USA) were added to the plates. Anti-CD4-BV605, anti-PD1-FITC, anti-CXCR5-APC, and anti-OX40-Percpcy 5.5 were added and labeled for 30 minutes. Then, after Fixations/Permeation Buffer (catalog no. 51-2092KZ, RRID: AB_2869009, BD, USA), Perm/Wash^TM^ buffer (catalog no. 51-2092KZ, RRID: AB_2869009, BD, USA) and anti-IL-21-BV421 (catalog no. 564755, RRID: AB_2738933) configured with Perm/Wash^TM^ buffer were respectively added; they were measured by flow cytometry.

### Purification of CD4^+^CXCR5^+^ Tfh Cells

Following the manufacturer's instructions, a REAlease CD4 Micro Beads kit (Miltenyi Biotec) was used to separate CD4^+^ T cells from PBMCs. After labeling with anti-CXCR5-APC for 30 minutes, CD4^+^CXCR5^+^Tfh cells were isolated from the purified CD4^+^ T cells after coculture with 20-L Anti-APC Micro Beads (Miltenyi Biotec) according to the manufacturer's instructions. The purity of the separated cells was monitored by flow cytometry. Purity >90% was acceptable.

### Coculture of Tfh and B Cells

We performed coculture assays using CD4^+^CXCR5^+^ Tfh cells and B cells that were separated from PBMCs by negative selection using a B-cell isolation kit (Miltenyi Biotec) at a ratio of 1:1 (1 × 10^5^:1 × 10^5^). The cocultures were incubated for 72 hours at 37 °C, 5% CO_2_, and 95% humidity. The concentrations of SEBAnti-CD3 mAb (200 ng/mL, clone: OKT3; BioCell), sOX40L (R&D, USA), and anti-OX40L (catalog no. MAB10541, RRID: AB_2272152) were 100, 200, 100, and 500 ng/mL, respectively. The experimental groups included B + Tfh, B + Tfh + sOX40L/anti-OX40L, and B cells alone. After 72-hour incubation, the cocultured cells were collected. After anti-CD19-BV605 and anti-CD138-APC were added and labeled for 30 minutes, they were measured by flow cytometry.

### Statistical Analysis

Graph Pad Prism 9.0 software (San Diego, CA, USA) was used for data analysis. The Shapiro–Wilk test was performed to determine data normality. To examine differences between the 2 groups, Student's *t*-tests (2-tailed) were performed. To assess the ratio of plasmacytes in the cocultured cells between the T1DM and T2DM groups, a 2-way ANOVA test was performed. Statistical significance was set at *P* < .05. Data are expressed as the mean ± SD.

### Ethics

This study was approved by the Biomedical Ethics Committee of the First Affiliated Hospital of Soochow University (2022180). All participants signed the informed consent forms.

## Results

### Clinical and Biochemical Characteristics of the Patients

Twenty-five patients with T1DM and 35 newly diagnosed T2DM were recruited. Their clinical and biochemical characteristics are shown in Table 1. The levels of fasting C-peptide and 2hCP were significantly lower in the T1DM group than in the T2DM group (*P <* .01), whereas the levels of GADA, ZnT8A, ICA, and IAA were significantly higher in the T1DM group (*P* < .01). There were no significant differences in the age, sex, fasting blood glucose, and HbA1c levels between the 2 groups (*P* > .05).

**Table 1.** Clinical and biochemical characteristics of the patients

	T2DM group (n = 35)	T1DM group (n = 25)	*P-*value
Age (year)	41.31 ± 2.45	36.432.95	.055
Male/female	25/10	14/11	.276
BMI (kg/m^2^)	29.29 ± 0.83	22.43 ± 0.90*^b^*	<.01
FBG (mmol/L)	8.90 ± 0.49	8.77 ± 0.95	.894
HbA_1c_ (%)	10.76 ± 0.38	10.99 ± 0.64	.745
FCP (ng/mL)	1.97 ± 0.21	0.46 ± 0.14*^b^*	<.01
2hCP (ng/mL)	5.26 ± 0.48	1.06 ± 0.25*^b^*	<.01
GADAb (IU/mL)	1.68 ± 0.34	83.36 ± 35.09*^b^*	<.01
ZnT8A (AU/mL)	0.60 ± 0.13	1.43 ± 0.59*^b^*	<.01
ICA (JDF units)	0.06 ± 0.00	0.79 ± 0.28*^b^*	<.01
IAA (JDF units)	0.17 ± 0.05	1.19 ± 0.48*^a^*	.011

Abbreviations: 2hCP, C-peptide after 2-hour meal; BMI, body mass index; FCP, fasting C-peptide; FPG, fasting plasma glucose; GADA, glutamic acid decarboxylase antibodies; HbA_1c_, glycosylated hemoglobin; IAA, insulin autoantibody; ICA, islet-cell antibodies; T1DM, type 1 diabetes mellitus; T2DM, type 2 diabetes mellitus; ZnT8A, zinc transporter-8 autoantibodies.

^*a*^ *P* < .05 compared with the control group.

^*b*^ *P* < .01 compared with the control group.

### Increased Circulating Tfh and IL-21^+−^ Tfh Cells in T1DM

**Figure 1.**
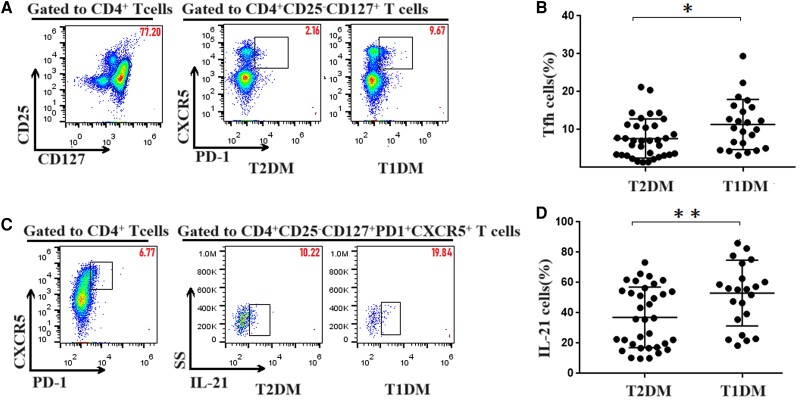
Flow cytometry analysis of the percentage of circulating Tfh cells and their IL-21 expressions in patients with T1DM and T2DM. PBMCs (5 × 10^5^/tube) were isolated from individual subjects and stained with anti-CD4, anti-CD25, anti-CD127, anti-PD1, anti-CXCR5, and intracellular anti-IL-21. The cells were characterized by flow cytometry analysis by gating initially on living lymphocytes and then on CD4^+^CD25^−^CD127^+^ effector T cells and CD4^+^CD25^−^CD127^+^CXCR5^+^PD1^+^ Tfh cells. Subsequently, the numbers of different subsets of Tfh cells and IL-21^+^ Tfh cells were calculated according to the frequency of effector T cells and Tfh cells. (A) Flow cytometry analysis of Tfh cells. (B) The numbers of CD4^+^CD25^−^CD127^+^CXCR5^+^PD1^+^ Tfh cells in T1DM and T2DM. A significant increase in the frequency of Tfh cells was observed in T1DM (11.35% ± 1.38%) compared to T2DM (7.63% ± 0.87%). (C) Flow cytometry analysis of IL-21^+^Tfh cells. (D) Percentage of IL-21^+^ Tfh cells in T1DM and T2DM. The IL-21 level is significantly higher in the T1DM group (53.08% ± 4.53%) than in the T2DM group (37.04% ± 3.37%). The experimental data are expressed as x ± SD. * *P* < .05; ** *P* < .01. Abbreviations: PBMC, peripheral blood mononuclear cell; T1DM, type 1 diabetes mellitus; T2DM, type 2 diabetes mellitus; Tfh, T follicular helper.

To measure the ratio of the Tfh-cell subset in T1DM, PBMCs from patients with T1DM and T2DM were detected by flow cytometry. The gating strategy for flow cytometric analysis of Tfh (CD4^+^CD25^−^CD127^+^CXCR5^+^PD1^+^) cells is shown in Fig. 1A. In contrast to T2DM (7.63% ± 0.87%), the percentage of circulating Tfh cells increased in T1DM (11.35% ± 1.38%), (*P* < .05, Fig. 1A and 1B). IL-21, produced by Tfh cells, is critical for GC formation as well as Tfh-cell generation. We further tested the expression of IL-21 in the Tfh cells. The expression of IL-21 was significantly higher in the T1DM group (53.08% ± 4.53%) than in the T2DM group (37.04% ± 3.37%) (*P* < .01, Fig. 1C and 1D). The results indicated that the elevated level of IL-21 may be associated with the increased ratio of the Tfh-cell subset in patients with T1DM.

### Upregulation of Tfh-cellular OX40 Expression in T1DM

**Figure 2.**
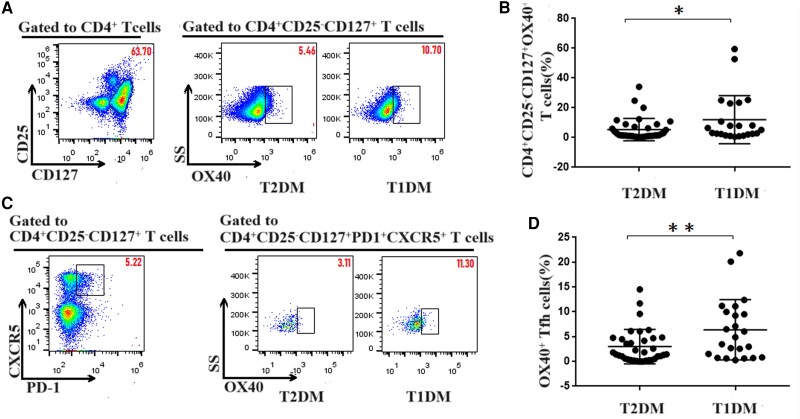
Flow cytometry analysis of the expression of OX40 in effector T and Tfh cells in patients with T1DM and T2DM. PBMCs (5 × 10^5^/tube) were isolated from individual subjects and stained with anti-CD4, anti-CD25, anti-CD127, anti-PD1, anti-CXCR5, and anti-OX40. The cells were characterized by flow cytometry analysis by gating initially on living lymphocytes and then on CD4^+^CD25^−^CD127^+^ effector T cells or CD4^+^CD25^−^CD127^+^CXCR5^+^PD1^+^ Tfh cells. Subsequently, the numbers of OX40^+^ effector T and OX40^+^ Tfh cells were calculated according to the frequency of effector T and Tfh cells. (A) Flow cytometry analysis of OX40^+^ effector T cells. (B) Number of OX40^+^ effector T cells in T1DM and T2DM. Compared with that in T2DM (5.42% ± 1.28%), OX40 expression in effector T cells increased in T1DM (12.02% ± 3.34%). (C) Flow cytometry analysis of OX40^+^ Tfh cells. (D) Numbers of OX40^+^ Tfh cells in T1DM and T2DM. The expression of OX40 was significantly higher in Tfh cells in the T1DM group (6.38% ± 1.29%) than in the T2DM group (2.98% ± 0.58%). The experimental data are expressed as x ± SD. * *P* < .05; ** *P* < .01. Abbreviations: PBMC, peripheral blood mononuclear cell; T1DM, type 1 diabetes mellitus; T2DM, type 2 diabetes mellitus; Tfh, T follicular helper.

To explore the role of OX40 in Tfh cells, the expression of OX40 in Tfh cells was further analyzed. The gating strategy for the flow cytometric analysis of OX40^+^ effector T cells and OX40^+^ Tfh cells is shown in Fig. 2. Compared with that in T2DM (5.42% ± 1.28%), the OX40 expression of effector T cells increased in T1DM (12.02% ± 3.34%) (*P* < .05, Fig. 2A and 2B). Moreover, the expression of OX40 in Tfh cells was significantly higher in T1DM (6.38% ± 1.29%) than in T2DM (2.98% ± 0.58%) (*P* < .01, Fig. 2C and 2D). Thus, OX40 was mainly expressed in Tfh cells.

### Upregulation of OX40L Expression in APCs in T1DM

**Figure 3.**
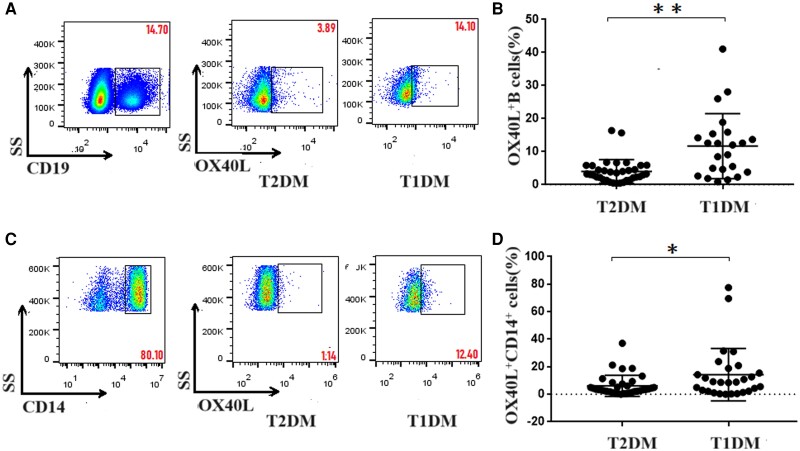
Flow cytometry analysis of the expression of OX40L in CD14^+^ monocytes and CD19^+^ B cells in T1DM and T2DM patients. PBMCs (5 × 10^5^/tube) were isolated from individual subjects and stained with anti-CD14, anti-CD19, and anti-OX40L. The cells were characterized by flow cytometry analysis by initially gating living monocytes and B cells. Subsequently, the numbers of OX40L^+^CD14^+^ monocytes and OX40L^+^CD19^+^ B cells were calculated. (A) Flow cytometry analysis of OX40L^+^CD19^+^ B cells. (B) Numbers of OX40L^+^CD19^+^ B cells in T1DM and T2DM. The percentage of OX40L^+^CD14^+^ monocytes (14.25% ± 3.59%) was higher in T1DM than in T2DM (6.12% ± 1.29%). (C) Flow cytometry analysis of OX40L^+^CD14^+^ monocytes. (D) Numbers of OX40L^+^CD14^+^ monocytes in T1DM and T2DM. The expression of OX40L in CD19^+^ B cells was higher in T1DM (11.68% ± 2.05%) than in T2DM (4.04% ± 0.60%). The experimental data are expressed as x ± SD. * *P* < .05; ** *P* < .01. Abbreviations: PBMC, peripheral blood mononuclear cell; T1DM, type 1 diabetes mellitus; T2DM, type 2 diabetes mellitus; Tfh, T follicular helper.

We further determined the expression of OX40L in CD14^+^ monocytes and CD19^+^ B cells in T1DM and T2DM. The percentage of OX40L^+^CD14^+^ monocytes was higher in T1DM (14.25% ± 3.59%) than in T2DM (6.12% ± 1.29%) (*P* < .05). The expression of OX40L in CD19^+^ B cells was higher in T1DM (11.68% ± 2.05%) than in T2DM (4.04% ± 0.60%) (*P <* .01). Overall, the expression of OX40L in APCs was obviously higher in T1DM than in T2DM. Therefore, we inferred that OX40L expressed in T1DM APCs might bind to OX40 expressed in Tfh cells to promote the differentiation and activation of Tfh cells (Fig. 3).

### Correlation of OX40^+^ Tfh With T1DM-related Antibodies

**Figure 4.**
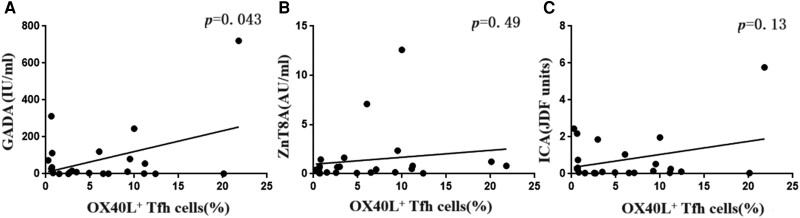
Correlation between serum levels of GADA, ZnT8A, and ICA and the ratio of OX40^+^ Tfh cells in T1DM. (A) Correlation of serum levels of GADA with the ratio of OX40^+^ Tfh cells in T1DM. (B) Correlation of serum ZnT8A levels with the ratio of OX40^+^ Tfh cells in T1DM. (C) Correlation of serum ICA levels with the ratio of OX40^+^ Tfh cells in T1DM. The percentage of OX40^+^ Tfh cells in T1DM is positively associated with GADA levels (*P* < .05) but not with ZnT8A and ICA levels. Abbreviations: GADA, glutamic acid decarboxylase antibodies; ICA, islet-cell antibodies; T1DM, type 1 diabetes mellitus; ZnT8A, zinc transporter-8 autoantibodies.

GADA, ZnT8A, ICA, and IAA, all of which are proteins associated with secretory granules in β-cells, are biomarkers of T1DM-associated autoimmunity and can be used to identify T1DM. Thus, the relationship between the percentage of OX40^+^ Tfh cells and the levels of serum T1DM-related antibodies, such as GADA, ZnT8A, and ICA, was further analyzed. We found that the percentage of OX40^+^ Tfh cells in T1DM was positively associated with the levels of GADA (*P <* .05) (Fig. 4A) but not with ZnT8A and ICA levels, suggesting that Tfh cells have a close relationship with GADA during the development of T1DM by OX40/OX40L signaling.

### Differentiation of B Cells Induced by Tfh Cells Through the OX40 Signal In Vitro

**Figure 5.**
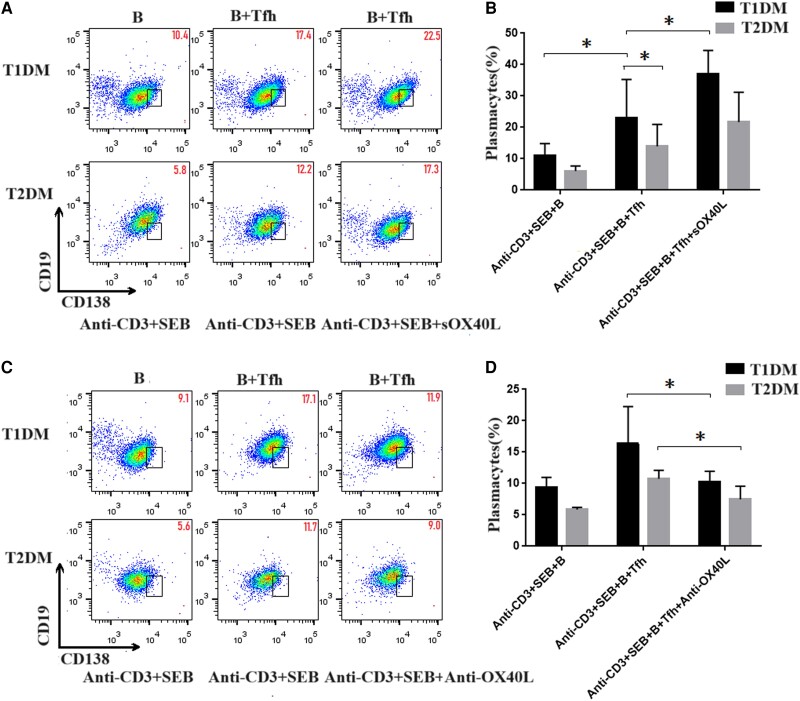
OX40 promoted B-cell differentiation induced by Tfh cells in vitro. Peripheral blood Tfh cells were obtained from 6 T1DM and 4 T2DM cells incubated with B cells with or without sOX40L. Cocultured cells were collected and stained with anti-CD19 and anti-138 antibodies. The cells were characterized by flow cytometry analysis by initially gating B cells. Subsequently, the numbers of CD19^−^CD138^+^ plasmacytes was calculated. The experimental groups included anti-CD3 + SEB + B, anti-CD3 + SEB + B + Tfh, and anti-CD3 + SEB + B + Tfh + sOX40L. (A) Flow cytometry analysis of CD19^−^CD138^+^ plasmacytes. (B) Percentages of plasmacytes in the different cocultured groups. Compared with T2DM, a higher percentage of plasmacytes was detected when B cells were cocultured with Tfh cells in T1DM. Furthermore, after sOX40L protein was added, the percentage of plasmacytes was significantly increased in the T1DM group. Blockade of OX40/OX40L signaling inhibits B-cell differentiation induced by Tfh cells in vitro. Peripheral blood Tfh cells obtained from 3 T1DM and 2 T2DM cells incubated with B cells with or without anti-OX40L antibodies. Subsequently, the numbers of CD19^−^CD138^+^ plasmacytes was calculated. Experimental groups included anti-CD3 + SEB + B, anti-CD3 + SEB + B + Tfh, and anti-CD3 + SEB + B + Tfh + anti-OX40L. (C) Flow cytometry analysis of CD19^−^CD138^+^ plasmacytes. (D) Percentages of plasmacytes in the different cocultured groups. Compared with the group without anti-OX40L antibodies, these antibodies significantly downregulated the percentage of plasmacytes in T1DM. Therefore, blockade of OX40/OX40L signaling inhibits B-cell differentiation induced by Tfh cells in vitro. The experimental data are expressed as x ± SD. * *P* < .05. Abbreviations: T1DM, type 1 diabetes mellitus; T2DM, type 2 diabetes mellitus; Tfh, T follicular helper.

To further identify the potential roles of Tfh cells in regulating B cells, peripheral B cells and Tfh cells from T1DM or T2DM were cocultured for 72 hours in vitro. Next, the ratio of CD19^−^CD138^+^ plasmacytes were analyzed by flow cytometry. As shown in Fig. 5A and 5B, there was a higher percentage of plasmacytes in T1DM than in T2DM. When B cells were cocultured with Tfh cells, the percentage of plasmacytes was upregulated. Furthermore, after sOX40L protein was added, the percentage of plasmacytes was significantly increased in the T1DM group (Fig. 5C and 5D). These data indicate that OX40 may have an important role in the differentiation of B cells by promoting Tfh-cell differentiation.

To further determine the potential role of OX40 during the differentiation of B cells stimulated by Tfh cells in vitro, anti-OX40L antibodies were added to the Tfh–B-cell coculture system for 72 hours. Compared with the group without anti-OX40L antibodies, these antibodies significantly downregulated the percentage of plasmacytes in T1DM. Therefore, blockade of OX40/OX40L signaling inhibited B-cell differentiation induced by Tfh cells in vitro. Together, these data indicated that OX40/OX40L may promote the differentiation of B cells stimulated by Tfh cells.

## Discussion

As an important member of the tumor necrosis factor receptor, the OX40 costimulatory molecule has an important role in promoting the proliferation and activation of CD4^+^ T cells. Kurata et al detected high expression of OX40 in Tfh cells, especially Tfh17 cells, in patients with rheumatoid arthritis (RA) and in RA mouse models (11). Jacquemin et al found that OX40/OX40L signaling was involved in the pathogenesis of systemic lupus erythematosus by promoting the differentiation of Tfh cells (12). However, the definite mechanism by which OX40/OX40L regulates Tfh cells in the pathogenic processes of T1DM remains unclear.

Our study indicated that abnormal differentiation of Tfh cells regulated by OX40 contributed to the immunopathological process in T1DM. Compared with patients with T2DM, patients with T1DM had larger numbers of OX40^+^ effector T cells, which was consistent with a previous finding (13), indicating that effector T cells were abnormally proliferated in patients with T1DM. Soroosh et al found that the number of effector T cells in OX40-knockout mice was significantly reduced, whereas the number of T central memory (TCM) cells remained normal (14), suggesting that OX40/OX40L has an important role in the accumulation of effector T cells. Furthermore, the results showed an increased number of CD4^+^CD25^−^CD127^+^CXCR5^+^PD1^+^ Tfh cells, IL-21^+^ Tfh cells, and OX40^+^ Tfh cells gated on CD4^+^CD25^−^CD127^+^ effector T cells in T1DM. Reportedly, Tfh cells promote the onset of T1DM through CXCR5 and/or IL-21. In peripheral lymphoid tissues, following activation by DCs, naïve T cells rapidly upregulate CXCR5 and arrive at the T–B-cell border under the chemotaxis of CXCL13 secreted by B cells and then enter the follicle to become Tfh cells, which assist naïve B cells to differentiate into plasma cells by secreting IL-21. The pathogenesis of T1DM is thought to involve T-cell and B-cell–mediated destruction of β-cells. The islet-targeting autoantibodies of GADA, ZnT8A, ICA, and IAA, all of which are proteins associated with secretory granules in β-cells, are biomarkers of T1DM-associated autoimmunity and can be used to identify and study individuals at risk of developing T1DM. Studies have demonstrated that B cells are crucial APCs in the pathogenic T-cell response to GADA and for overcoming a checkpoint in T-cell tolerance to B-cell antigens. Although proinflammatory Tfh cells are considered to be the functional mediators of cell destruction in the pancreatic islets, the exact role of B cells in the disease process of T1DM remains unclear (15-19). More importantly, the increased number of OX40^+^ Tfh cells in patients with T1DM was positively associated with the level of GADA, a marker of autoantibodies for T1DM. Glutamic acid decarboxylase (GAD) is an intracellular enzyme fairly expressed in insulin-secreting pancreatic β cells and neurons. The physiologic function of GAD is decarboxylation of glutamate to gamma-aminobutyric acid (20-23). Responses to immunodominant GAD65 peptides were also absent in B-cell–deficient nonobese diabetic mice, suggesting that B cells are crucial for the autoimmune response in T1DM. Therefore, OX40^+^ Tfh cells are closely associated with the immunopathological process of T1DM. In addition, flow cytometry analysis demonstrated a significant increase in OX40L expression in CD14 monocytes and CD19 B cells in T1DM, consistent with the trend of OX40 expression. Thus, OX40 molecules expressed in Tfh cells may combine with OX40 ligands expressed in APCs to influence Tfh differentiation and function and induce the development of T1DM.

To further define the function of OX40 in Tfh cells in T1DM, a Tfh–B-cell coculture system was used in vitro. The ratio of CD19^−^CD138^+^ plasmacytes increased when B cells were cocultured with Tfh cells isolated from T1DM. Adding sOX40L protein further increased the proportion of CD19^−^CD138^+^ plasmacytes, which indicated that OX40/OX40L signaling could enhance Tfh-cell function and promote B-cell activation and antibody secretion. Furthermore, the OX40/OX40L pathway was blocked by anti-OX40L antibodies in the Tfh–B-cell coculture system, and an obviously decreased ratio of CD19^−^CD138^+^ plasmacytes was detected, which indicated inhibition of B-cell differentiation when OX40 signaling was weakened. These data suggest that the mechanisms of B-cell activation and differentiation induced by OX40^+^ Tfh cells exist in 2 aspects. One aspect is the combination of OX40 expressed in Tfh cells and OX40L expressed in B cells, which delivers signaling to enhance the function of Tfh cells and production of IL-21, which in turn induces plasma cell differentiation. The other aspect is reverse signaling, which is delivered from OX40 expressed in Tfh cells to OX40L expressed in B cells to promote B-cell differentiation directly. In summary, OX40/OX40L signaling between Tfh cells and B cells is bidirectional, which could affect B cells directly or indirectly by enhancing Tfh function.

Previous studies have suggested that Tfh cells have an important role in the pathogenesis of various autoimmune diseases, such as T1DM, systemic lupus erythematosus, and RA. These diseases result from the inability of the immune system to suppress autoreactive T-cell responses and autoantibody production (24-27). Thus, the main function of Tfh cells is to assist antigen-specific B-cell proliferation and antibody classification conversion and promote the differentiation of B lymphocytes into plasmacytes and memory B cells. Tfh cells assist B cells mainly by secreting cytokines and by direct contact in the GC. IL-21, produced by Tfh cells, can induce B cells to differentiate into plasmacytes, which can secrete antibodies (28-30). Tfh cells assist B cells, which are indispensable for high-affinity antibody production and memory B-cell yield.

In summary, our study demonstrated that the function of Tfh cells and B cells is regulated by the OX40/OX40L axis in T1DM. However, there are still some problems related to this issue that remain unresolved in this study. Given that blocking OX40/OX40L signaling has previously shown great therapeutic effects in some mouse models of autoimmune diseases, targeting OX40/OX40L is promising as a new therapeutic approach for these diseases.

Rituximab, an anti-CD20 monoclonal antibody, represses Tfh differentiation by decreasing IL-21, which inhibits the differentiation of Tfh cells by distinct mechanisms and thus exert therapeutic effect on T1DM. Rocatinlimab, an anti-OX40 antibody, was evaluated in a clinical trial in patients with atopic dermatitis; however, its therapeutic effect on T1DM is unknown. The efficacy data from clinical trials that targeted OX40/OX40L and T1DM are limited. In addition, the exact mechanism by which the OX40/OX40L axis affects Tfh also remains unknown at present. Therefore, additional studies are needed to evaluate the biological characteristics of these cells and their mechanisms involved in the prevention or treatment of T1DM and other autoimmune diseases.

## Conclusion

Compared with patients with T2DM, the proportion of peripheral Tfh cells increased and the expression of OX40 in peripheral Tfh cells was upregulated in patients with T1DM. The OX40/OX40L signal can enhance the function of Tfh cells to assist B-cell differentiation and autoantibody production, a finding that potentially can be used in new approaches for preventing or treating T1DM.

## Data Availability

All datasets generated during and analyzed during the current study are not publicly available but are available from the corresponding author on reasonable request.
